# Induction of apoptosis by a potent caffeic acid derivative, caffeic acid undecyl ester, is mediated by mitochondrial damage in NALM-6 human B cell leukemia cells

**DOI:** 10.3892/or.2012.2163

**Published:** 2012-12-03

**Authors:** AYAKO TOMIZAWA, SYU-ICHI KANNO, YUU OSANAI, AKANE GOTO, CHIZURU SATO, SHIN YOMOGIDA, MASAAKI ISHIKAWA

**Affiliations:** Department of Clinical Pharmacotherapeutics, Tohoku Pharmaceutical University, Sendai, Japan

**Keywords:** caffeic acid, apoptosis, Bcl-2, mitochondria, NALM-6

## Abstract

Caffeic acid esters have various biological activities, and we previously reported that undecyl caffeate (caffeic acid undecyl ester, CAUE), a new caffeic acid derivative, has strong pharmacological activity. The present study investigated the cytotoxicity of both CAUE and its parent compound, caffeic acid phenethyl ester (CAPE), and characterized the mechanisms by which they induce apoptosis in the human B cell leukemia cell line NALM-6. Treatment with CAUE reduced cell survival in NALM-6 cells but had no significant effect on the survival of normal lymphocytes. When assessing the 50% inhibitory concentration (IC_50_) for cytotoxicity, CAUE had 10-fold higher activity than CAPE in NALM-6 cells. CAUE treatment resulted in induction of apoptotic features in NALM-6 cells, including cleaved poly (ADP-ribose) polymerase and activated caspase-3. A caspase inhibitor completely blocked CAUE-induced apoptosis. CAUE treatment resulted in a concentration- and time-dependent decrease in both mitochondrial membrane potential and downregulation of Bcl-2 expression. Moreover, CAUE-induced apoptosis was enhanced in the Bcl-2 knockdown condition induced by small interfering RNA. These data suggest that CAUE-induced apoptosis was mediated via an apoptotic intrinsic pathway including mitochondrial damage and was caspase-dependent. These data also suggest that CAUE is a powerful anti-leukemic agent that acts via induction of apoptosis by mitochondrial damage and selective action in leukemia cells.

## Introduction

Caffeic acid esters are a component of propolis and exert various biological activities (1), such as antioxidant (2) and anti-inflammatory effects (3). Caffeic acid derivatives, such as caffeic acid phenethyl ester (CAPE), have anticancer effects (4). Previous studies have indicated that CAPE exerts cytotoxicity via induction of apoptosis through caspase activation and mitochondrial-mediated pathways (5,6). Apoptosis induced by mitochondrial damage involves a decrease in the permeability of the mitochondrial membrane and is directly regulated by the Bcl-2 protein (7). Downregulation of the Bcl-2 protein might be a useful method to modulate apoptosis and thereby increase the chemotherapeutic effect of anticancer drugs (8).

We previously demonstrated that octyl caffeate, a compound semi-derived from caffeic acid, was 6-fold more potent that CAPE in terms of cytotoxicity against human leukemia U937 cells (9). Further, previous studies have demonstrated that undecyl caffeate (caffeic acid undecyl ester, CAUE), a new caffeic acid derivative, was a highly potent inhibitor of lipopolysaccharide-induced nitric oxide production in RAW 264.7 mouse macrophage cells (10). However, the cytotoxic effects and apoptotic mechanisms by which CAUE act remain unclear. Therefore, the goal of the present study was to investigate the cytotoxicity of CAUE and its parent compound, CAPE, and to characterize the mechanisms by which they induce apoptosis in the human B cell leukemia cell line NALM-6.

## Materials and methods

### Materials and cell culture

CAUE were prepared as previously described (10). CAPE and all other reagents, unless stated, were of the highest grade available and were purchased from either Sigma (St. Louis, MO, USA) or Wako Pure Chemical Industries, Ltd. (Osaka, Japan). Normal human lymphocytes were provided by healthy volunteers and prepared by Ficoll-Paque™ PLUS (Amersham, Arlington Heights, IL, USA), according to manufacturer’s protocol. Research protocols were approved by the Ethics Committee of Tohoku Pharmaceutical University. Human B cell leukemia NALM-6 cells were supplied by the Cell Resource Center for Biomedical Research, Tohoku University (Sendai, Japan). All cell culture reagents and small interfering RNAs (siRNAs) were obtained from Invitrogen Corp. (Carlsbad, CA, USA). Cells were routinely cultured using standard methods as described in our previous studies (11,12).

### MTT assay

Cytotoxicity was assessed by the MTT [3-(4, 5-dimethylthiazol-2-yl)-2, 5-diphenyl tetrazolium bromide] assay, with slight modification of our previously described method (13). Briefly, cells were seeded from 1 to 4×10^4^ cells in 96-well plates. Cells were incubated with CAUE at indicated concentrations for 24 to 72 h, followed by the addition of 10 μl of MTT (5 mg/ml saline) to each well. The sample was incubated for 90 min at 37°C, the supernatant was aspirated, and the cells were lysed and solubilized by the addition of 100 μl of 0.04 N HCl in isopropanol. The absorbance of each well was determined at 590 nm using an Inter-med model NJ-2300 Microplate Reader. Control cells were treated with 0.5% dimethyl sulfoxide (DMSO) as CAUE vehicle. The viability of cells was calculated by the following formula: absorbance in treated sample/absorbance in control ×100%.

### Assessment of apoptosis

Detection of apoptotic cells was estimated by nuclear morphological observation and performed by flow cytometry using a FACSCalibur flow cytometer (Becton Dickinson, San Jose, CA, USA). CellQuest Pro software (Becton Dickinson) was then used to analyze hypodiploid cells (apoptotic sub-G1 peak), with slight modification of our previously described method (11).

### Western blotting

The effects of cellular signal transduction on protein expression by CAUE-induced apoptosis or confirmation of Bcl-2 siRNA knockdown assay were estimated by western blotting (13). Briefly, after incubation of cells with the indicated concentration of CAUE, the cells were washed with phosphate buffered saline (PBS) and lysed. The protein concentration was measured by the BCA™ protein assay kit (Thermo Fisher Scientific, Inc. Rockford, IL, USA), according to the instructions provided by the manufacturer. Samples of each protein (30 μg) were loaded onto a 10% sodium dodecyl sulfate (SDS)-polyacrylamide gel. After electrophoresis, the protein was transferred to a polyvinylidene difluoride (PVDF) membrane. The protein was blocked with blocking solution (25 mM Tris-HCl, pH 7.4, 137 mM NaCl, 2.68 mM KCl and 5% skim milk) for 1 h and incubated with antibody overnight at 4°C. The membrane was then washed with blocking solution without skim milk and incubated with horseradish peroxidase-linked secondary antibody for 1 h. After washing again with wash buffer, protein levels were analyzed by enhanced chemiluminescence with an ECL Western Blotting Detection system (Amersham). All antibodies used were purchased from Cell Signaling Technology, Inc. (Beverly, MA, USA).

### Measurement of the mitochondrial membrane potential

After CAUE treatment, NALM-6 cells were incubated with 0.3 μM rhodamine 123 (R123) for 15 min at 37°C. Cells were then washed with PBS and collected by centrifugation and the collected cells were suspended in 500 μl of PBS. Fluorescence intensities of R123 were analyzed on a FACSCalibur flow cytometer set at 488 nm excitation (FL1 blue laser).

### Bcl-2 siRNA knockdown assay

siRNA-Bcl-2 (siBcl-2) and siRNA-control [as non-targeting siRNA; negative control (Neg)] were transfected into NALM-6 cells using the Neon™ Transfection System (Invitrogen Corp.) according to the instructions provided by the manufacturer. Briefly, 40–60% confluent cells were harvested and then washed twice with PBS. Then, 2×10^5^ cells containing 50 pmol of each siRNA in 10 μl Neon tip were electroporated at 1380V, pulse length of 10 msec, and 3 pulse times performed. After culture overnight without antibiotics, samples were used for assessment of Bcl-2 expression or detection of CAUE-induced apoptosis. The mRNA level of Bcl-2 (GenBank accession no. NM_000633.2) was quantified using the real-time polymerase chain reaction (qPCR) with a Light Cycler (Roche, Basel, Switzerland). Briefly, total RNA was extracted from each cell line with the Isogen reagent (Nippon Gene, Tokyo, Japan), and 0.1 μg of total RNA was then reverse transcribed to single-strand cDNA using the ReverTra Ace^®^ qPCR RT kit (Toyobo, Osaka, Japan). Aliquots of the cDNA preparations were subjected to qPCR analysis using SYBR^®^ Premix Ex Taq™ (Takara Bio, Shiga, Japan) to quantify the expression of each target gene and the internal standard, β-actin (GenBank accession no. NM_001101.3), using Light Cycler. The Takara Perfect Real- time Primers (Takara Bio) were used as primer pairs. The results of all assays were checked against melting curves to confirm the presence of single PCR products. Knockdown of Bcl-2 protein was validated by western blotting, as described above.

### Statistical analysis

Statistical analysis was performed using a one-way analysis of variance (ANOVA) followed by the Williams’ type multiple comparison test or a Bonferroni test among multiple groups. A p-value of <0.01 was considered significant.

## Results

### Cytotoxic effect of CAUE

**Figure 1:**
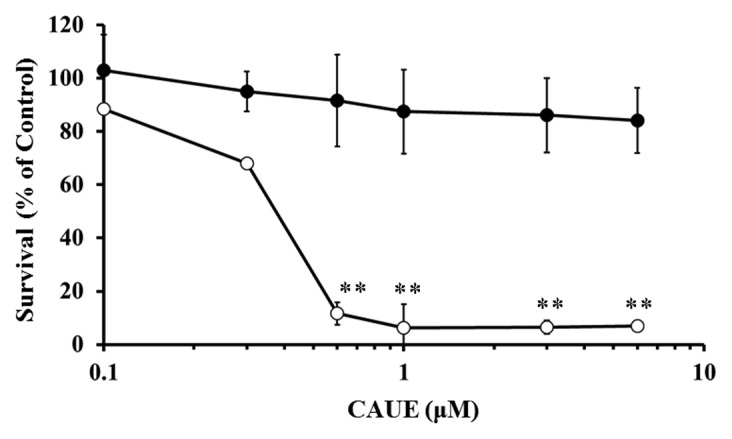
Cytotoxicity of undecyl caffeate (caffeic acid undecyl ester, CAUE) against normal human lymphocytes or B cell leukemia NALM-6 cells. Closed (●) or open circle (○) indicates normal human lymphocytes or NALM-6 cells, respectively. Cytotoxicity was assessed using the MTT assay following incubation of the cells with the indicated concentration of CAUE for 24 h. Survival (%) was calculated relative to the each control (CAUE vehicle). The results are means ± SEM of three individual studies. ^**^p<0.01 compared with the control group under the indicated culture condition.

First, we examined the cytotoxic effect of CAUE incubation for 24 h on normal human lymphocytes or B cell leukemia NALM-6 cells via the MTT assay (Fig. 1). CAUE treatment in concentrations >0.3 μM resulted in marked reduction in cell survival of NALM-6 cells but had no significant effect on normal lymphocytes, even at 6 μM. The 50% inhibitory concentration (IC_50_) of CAUE incubation for 24 and 72 h on NALM-6 was 0.33 and 0.16 μM, respectively. The IC_50_ of incubation with CAPE for 24 and 72 h in NALM-6 cells was 5.39 and 1.74 μM, respectively. Thus, CAUE was a more potent cytotoxic agent than CAPE on NALM-6 cells, yet did not exert a cytotoxic effect on normal cells.

### Induction of apoptotic cells by CAUE

**Figure 2:**
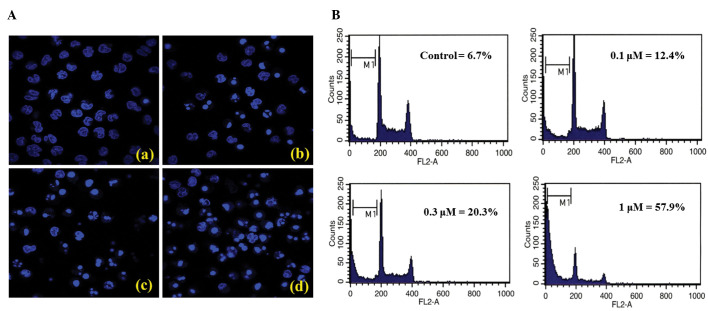
Induction of apoptotic phenotype by CAUE in NALM-6 cells. (A) Nuclear morphological changes. NALM-6 cells were treated with 1 μM CAUE for 0 (a), 6 (b), 12 (c), or 24 h (d). Cell nuclei were then stained with H33342 and evaluated by fluorescent microscopy (magnification, ×400). (B) Detection of hypodiploid cells (sub-G1 peak). Incubation with indicated concentration of CAUE for 12 h, with subsequent measurement of the apoptotic sub-G1 peak by flow cytometry. Experiments are representative of a minimum of three separate experiments.

**Figure 3:**
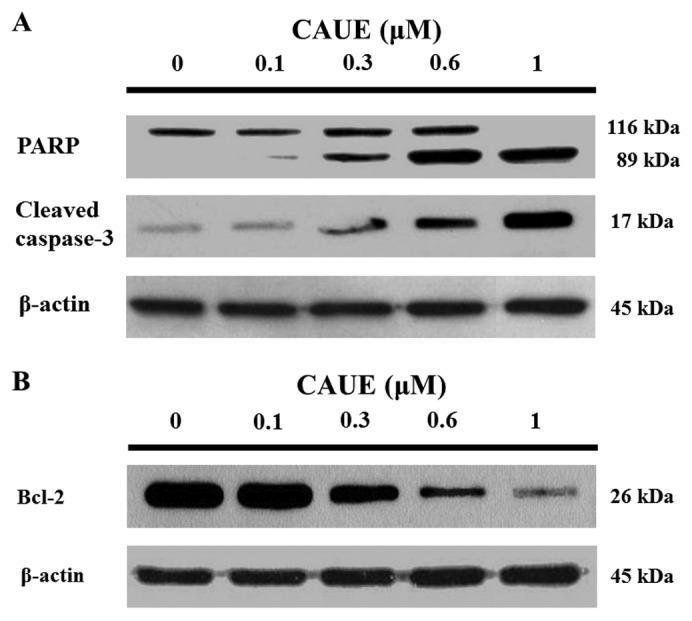
Western blotting analysis of CAUE-induced changes in the level of apoptosis related proteins. Cells were incubated with indicated concentration of CAUE overnight (A) or for 6 h (B), and expression of the indicated proteins was then analyzed by western blotting using expression of β-actin as a loading control. Experiments are representative of a minimum of three separate experiments.

**Figure 4:**
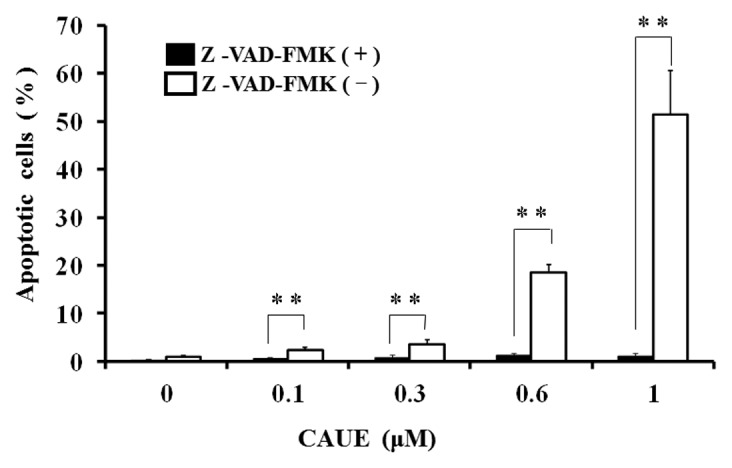
Effects of caspase inhibitor on CAUE-induced apoptosis. Cells were pretreated with the caspase inhibitor, Z-VAD-FMK (40 μM), for 1 h and then incubated with the indicated concentration of CAUE overnight. Apoptotic cells were then detected by indicating nuclear morphological change stained with H33342. The results are means ± SEM of three individual studies. ^**^p<0.01 compared with or without incubation with Z-VAD-FMK under each indicated CAUE concentration.

Next, we examined whether CAUE caused apoptosis of NALM-6 cells by assessing nuclear morphological change and the presence of hypodiploid cells (sub-G1 peak) using flow cytometry. Overnight incubation of NALM-6 cells in CAUE produced a concentration-dependent increase in the typical phenotype of apoptosis, including nuclear chromatin condensation, apoptotic bodies (Fig. 2A) as well as an increase in hypodiploid cells (Fig. 2B). Further, western blotting demonstrated concentration-dependent cleavage of poly (ADP-ribose) polymerase and caspase-3 protein in response to CAUE (Fig. 3A). Z-VAD-FMK, a broad spectrum caspase inhibitor, completely inhibited CAUE-induced apoptosis (Fig. 4), which indicated that CAUE induced apoptosis in a caspase-dependent manner.

### Mitochondrial damage by CAUE and effect of Bcl-2 knockdown

**Figure 5:**
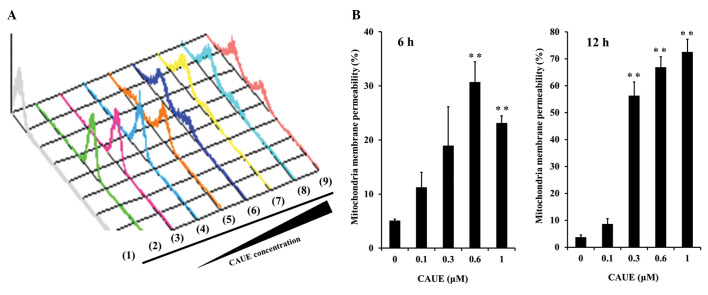
Effects of CAUE on mitochondrial membrane potential. Mitochondrial membrane potential was assessed via uptake of R123, a substrate of mitochondrial membrane permeability, on flow cytometry assay. (A) A typical histogram of data showing a decrease in uptake of R123 after incubation with CAUE for 12 h, no incubation with R123 (1; gray), a single incubation with 0.3 μM R123 (2; green), and CAUE at 0 (3; magenta), 0.1 (4; blue), 0.3 (5; orange), 0.6 (6; dark blue), 1 (7; yellow), 3 (8; light blue), or 6 μM (9; pink). Experiments are representative of a minimum of three separate experiments. (B) Quantitative results of mitochondrial membrane permeability indicating fluorescent intensity of R123 uptake. The results are means ± SEM of three individual studies. ^**^p<0.01 compared with the control group under the indicated culture condition.

**Figure 6:**
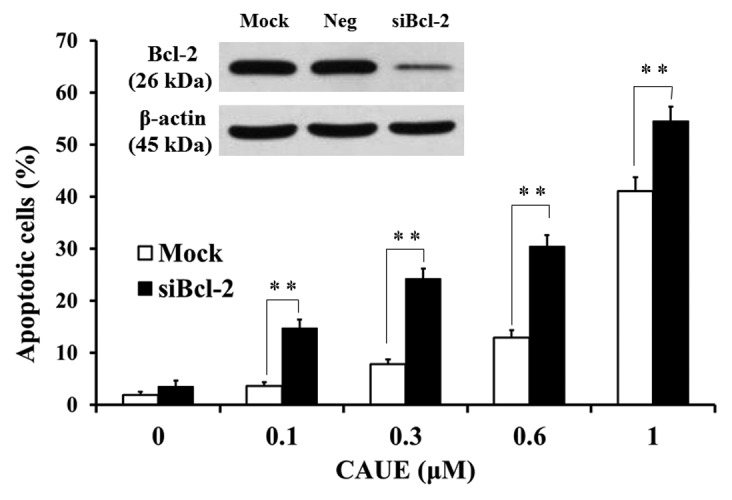
The effects of Bcl-2 knockdown on CAUE-induced apoptosis in NALM-6. Either siRNA-Bcl-2 (siBcl-2) or siRNA-control as non-targeting siRNA (Negative control: Neg) were transfected into NALM-6 cells using electroporation, as described in Materials and methods. The mock group was not transfected with siRNAs, but did undergo electroporation. (Inset) The expression of Bcl-2 protein was analyzed by western blotting, using expression of β-actin as a loading control. Experiments shown are representative of a minimum of three separate experiments. The results are means ± SEM of three individual studies. ^**^p<0.01 compared with each mock group.

To characterize the involvement of mitochondrial damage in CAUE-induced apoptosis in NALM-6 cells, we used western blotting to analyze Bcl-2 protein and a flow cytometry assay to analyze mitochondrial membrane potential by uptake of R123, a substrate of mitochondrial membrane permeability. Bcl-2 protein levels were downregulated in a concentration-dependent manner after incubation with CAUE for 6 h (Fig. 3B). Likewise, incubation with CAUE for 6 h significantly reduced mitochondrial membrane potential, and this effect was potentiated by incubation with CAUE for 6 h (Fig. 5A and B). In response to siRNA of Bcl-2 (siBcl-2), Bcl-2 mRNA levels in NALM-6 cells were reduced to 15% of levels seen in cells that underwent mock treatment, while there was no change in Bcl-2 mRNA levels in the negative control group (Neg). Further, Bcl-2 protein levels were downregulated in response to siBcl-2, but did not change in the mock or Neg group (Fig. 6, inset). CAUE significantly potentiated the induction of apoptosis in cells treated with siBcl-2 when compared with the mock group (Fig. 6). These data suggest that induction of apoptosis by CAUE was mediated by mitochondrial damage and Bcl-2 downregulation.

## Discussion

Chemotherapy is a powerful tool for the treatment of various cancers, including hematologic malignancies. In particular, lymphoblastic leukemia is highly aggressive, but frequently curable with current therapy (14). The NALM-6 cell line was originally established from the peripheral blood of a patient with acute lymphoblastic leukemia (15). We employed this cell line in the present study and assessed the cytotoxic effect of CAUE, a new caffeic acid derivative, to determine its potential utility as a treatment for lymphoblastic leukemia. As shown in Fig. 1, CAUE exerted concentration-dependent cytotoxicity in NALM-6 cells but did not affect normal lymphocytes. The cytotoxic action of the parental compound of CAUE, CAPE, has been investigated in tumor cells but not in normal cells (16,17). The present data suggest that CAUE and CAPE have similar biologic activity, although CAUE was more potent than CAPE, as demonstrated by its 10-fold higher cytotoxic IC_50_. These results therefore suggest that CAUE is a powerful chemotherapeutic reagent and has selective action on leukemia cells.

Apoptosis is an important mechanism by which anticancer drugs induce an antitumor response (18). The core effectors of apoptosis encompass proteolytic enzymes of the caspase family, which reside as latent precursors in most nucleated metazoan cells (19). A majority of studies on apoptosis are based on the assumption that caspase precursors are activated by cleavage, a common mechanism for most protease zymogen activations. Nuclear poly (ADP-ribose) polymerase (PARP) is one of the main cleavage targets of caspase-3 and appears to be involved in DNA repair in response to environmental stress (20). Full length PARP (116 kDa) cleave to a specific 89 kDa form observed during apoptosis (21). As shown in Figs. 2 and 3, treatment with CAUE resulted in induction of apparent apoptotic features in NALM-6 cells, including cleaved PARP and activated caspase-3. Further, caspase inhibitor completely blocked induction of apoptosis by CAUE (Fig. 4). These results suggest that activation of caspase plays a pivotal role in CAUE-induced apoptosis in NALM-6.

Apoptosis involves two major pathways, the intrinsic and extrinsic pathway. The intrinsic pathway involves mitochondria as initiators of cell death. In this study, the cytotoxic effect of CAUE was estimated by MTT assay as a reflection of mitochondrial activity (Fig. 1). It is reasonable to assume that CAUE-induced apoptosis involves a decrease in mitochondrial activity. To characterize the apoptotic mechanism by CAUE, the present study focused on the intrinsic pathway of apoptosis induction, which involves mitochondrial damage. As shown in Fig. 5, CAUE induced a concentration- and time-dependent decrease in mitochondrial membrane potential. Induction of apoptosis by CAUE has showed incubation following 6 h (Fig. 2A-b). Incubation with CAUE for 6 h also downregulated Bcl-2 expression (Fig. 3B). These results suggest that CAUE-induced apoptosis was caused by mitochondrial damage. As shown in Fig. 6, CAUE-induced apoptosis was enhanced in the Bcl-2 knockdown condition induced by siRNA. Several randomized, controlled, phase III trials have evaluated the utility of antisense Bcl-2 combined with standard chemotherapy for the treatment of patients with chronic lymphocytic leukemia, multiple myeloma, malignant melanoma, or non-small cell lung carcinoma (8). However, attempts to target Bcl-2 therapeutically using antisense technology to inhibit protein translation have not significantly improved outcomes for cancer patients, although improved oligonucleotide design may potentially enhance the efficacy of this approach (22). Various Bcl-2 inhibitors for anti-apoptotic Bcl-2 proteins are markedly different in terms of potency and selectivity (23). The present results suggest that CAUE is a potent inhibitor of Bcl-2 and may be an effective chemotherapy for leukemia.

In conclusion, CAUE has a potent cytotoxic effect in NALM-6 cells but not on normal lymphocytes, CAUE-induced apoptosis is mediated by mitochondrial damage and caspase. Thus, CAUE may be an effective chemotherapy for leukemia, and decreases in Bcl-2 expression via co-treatment with other chemotherapeutical reagent may enhance the chemotherapeutic action of CAUE on leukemia patients.
